# Sensory-motor training targeting motor dysfunction and muscle weakness in long-term care elderly combined with motivational strategies: a single blind randomized controlled study

**DOI:** 10.1186/s11556-016-0164-0

**Published:** 2016-05-28

**Authors:** Slavko Rogan, Lorenz Radlinger, Heiner Baur, Dietmar Schmidtbleicher, Rob A. de Bie, Eling D. de Bruin

**Affiliations:** Discipline Physiotherapy, Health, Bern University of Applied Sciences, Bern, Switzerland; Department of Sport Science, Wolfgang-Goethe University Frankfurt, Frankfurt, Germany; Department of Epidemiology, Maastricht University, CAPHRI School for Public Health and Primary Care, Maastricht, The Netherlands; Centre for Evidence Based Physiotherapy, Maastricht University, PO Box 616, 6200 MD Maastricht, The Netherlands; Department of Health Sciences and Technology, Institute of Human Movement Sciences and Sport, ETH Zurich, Switzerland Wolfgang-Pauli-Str. 27, HIT J 31.2, CH-8093 Zurich, Switzerland

**Keywords:** Sensory-motor training, Motor dysfunction, Muscle weakness, Long-term care, Motivational strategies

## Abstract

**Background:**

This study evaluated the effects of a combined innovative training regime consisting of stochastic resonance whole-body vibration (SR-WBV) and a dance video game (DVG) on physical performance and muscle strength in long-term-care dwelling elderly.

**Methods:**

Thirthy long-term-care elderly were randomly allocated to an intervention group (IG; *n* = 16) receiving combined SR-WBV training and DVG, or a sham group (SG; *n* = 14). IG performed five sets one minute of SR-WBV, with one minute rest between sets (base frequency 3 Hz up to 6 Hz, Noise 4) during the first five weeks on three days per week. From week five to eight a DVG was added to SR-WBV for IG on three days per week. SG performed a five-set SR-WBV program (1 Hz, Noise 1) lasting five times one minute, with one minute rest in between, three days a week. From week five to eight stepping exercises on a trampoline were added on three days per week. Primary outcome: Short physical performance battery (SPPB). Secondary outcome: isometric maximal voluntary contraction (IMVC), and sub phases of IMVC (Fsub), isometric rate of force development (IRFD) and sub time phases of IRFD (IRFDsub) were measured at baseline, after four and eight weeks. ANOVA with repeated measures was used for analyses of time and interaction effects and MANOVA determined between group intervention effects.

**Results:**

Between group effects revealed significant effects on the SPPB primary outcome after four weeks F(1, 27) = 6.17; *p* = 0.02) and after eight weeks F(1,27) = 11.8; *p* = 0.002). Secondary muscle function related outcome showed significant between group effects in IG on IRFD, Fsub 30 ms, 100 ms, 200 ms and IRFDsub 0-30 ms, 0-50 ms, 0-100 ms and 100-200 ms compared to SG (all *p* < 0.05).

**Conclusions:**

Eight weeks SR-WBV and DVG intervention improved lower extremity physical function and muscle strength compared to a sham intervention in long-term-care elderly. SR-WBV and DVG seems to be effective as a training regime for skilling up in long-term-care elderly.

**Electronic supplementary material:**

The online version of this article (doi:10.1186/s11556-016-0164-0) contains supplementary material, which is available to authorized users.

## Background

‘The competence of an individual to have the physiological capacity to perform normal everyday activities safely and independently without undue fatigue’ [1] signifies the functional abilities of an individual. Disability, defined as difficulty or dependency in the execution of the activities of daily living, is associated with increased healthcare utilization and related costs [2]. Disability in frail older people is considered a public health problem [3] in which prevention has to be considered a priority for research and clinical practice [4]. Physical activity (PA) for the elderly is one of the major elements for general health prevention [5]; therefore inactive or sedentary elderly should increase their PA [6]. Despite the known benefits of PA, residents living in long-term care (LTC) are relatively sedentary [7, 8].

The loss of muscle mass and strength with age, coined sarcopenia, is recognized as a major cause of disability and morbidity in the elderly [9]. Sarcopenia describes the progressive decline in skeletal muscle mass and function (strength or performance) with advancing age [10]. However, recent studies demonstrated that muscle atrophy is a relatively small contributor to the loss of muscle strength [11–13]. Changes in neurologic function and/or the intrinsic force-generating properties of skeletal muscle are recently proposed to be responsible for muscle weakness and motor dysfunction in the elderly [11, 14–18]. Dynapenia has been used to coin this age-associated loss of muscle strength and power with its significant clinical consequences; e.g., the increased risk for functional limitations, disability, and mortality. Dynapenia encompasses broader aspects of skeletal muscle performance, and so includes strength (i.e., maximal voluntary force) and/or mechanical power (a product of force - time velocity) [19] together with aspects of neurological functioning [20, 21].

Both neurologic and skeletal muscle properties are necessary for optimal muscle force production and control [16, 17, 22]. The nervous system’s ability to fully activate a skeletal muscle voluntarily for example seems to be impaired in individuals with dynapenia [23]. Furthermore, poor sensorimotor nerve function independently predicts mobility disability [24]. Targeting neural structures through exercise is, therefore, considered important in influencing muscle strength in elderly [25].

Efficient movement function and the maintenance of balance function during dynamic tasks; e.g., during walking, are more complex than merely adequate force production from the muscles [26]. For whole body movements it is important to precisely coordinate muscle actions. This requires sensory, biomechanical and motor-processing strategies along with learned responses from previous experiences and anticipation of change [27, 28]. Adequately combining three levels of motor control (spinal reflex, brain stem balance, and cognitive programming) produces appropriate muscle responses [29]. So, from these results, it can be hypothesised that when focusing on these three levels of motor control in a training program there will be improvements in muscle recruitment and timing and, hence, physical functioning and muscle strength.

Stochastic resonance (SR) is a phenomenon in nonlinear systems characterized by a response increase of the system induced by a particular level of input noise [30, 31]. One of the first studies applying noise in humans revealed increased sensitivity to detect sub-threshold tactile stimuli as an effect to such an intervention [32]. Cordo and colleagues [33] were among the first to demonstrate that the application of noise on human muscle spindle receptors improved afferents sensitivity in the human motor system and suggested, based on their results, that a stochastic-resonance based technique could be applied in clinical settings to individuals with elevated cutaneous thresholds; e.g., to older adults [33]. First evidence that mechanical noise applied to the feet via vibrating insoles improved balance in standing position stems from Pripatla et al. [34] and Collins and co-workers [35]. Systematic reviews concluded that, compared to more demanding interventions, whole-body vibration (WBV) as a sensorimotor training might be a safer and less fatiguing type of exercise [36] with a beneficial effect on movement skills [37] and muscle strength [38]. Stochastic resonance whole-body vibration (SR-WBV) has been described as stimulating sensorimotor processes [31, 39] with a positive effect on muscle functional strength [38]. The SR-WBV stimulus triggers muscle spindles and, thereby, improves the functionality of the muscle-nerve system [40] and adjusts afferent and efferent signals which, in turn, are leading to “training” effects for the sensorimotor system [41]. Muscle strength increase following SR-WBV is mainly attributed to neural adaptation bringing on improvements in inter- and intra-muscular coordination [42]. See [43] for an overview. Virtual reality training techniques may be used to incorporate cognitive programming elements into exercise [44] and could, hence, also be part of a training program for elderly [45]. Pilot trials with long term care dwelling elderly showed beneficial effects on physical performance for those adhering to an SR-WBV intervention, however, the program requires modifications that target improved compliance with the intervention [46]. Interventions performed with frail individuals often suffer from low adherence rates and are, therefore, advised to specifically include support and motivation strategies, as well as giving assistance to individuals to develop both goals and the strategies to achieve these [47, 48].

The aim of this study was to assess the effects of a sensorimotor training program with SR-WBV & Virtual Reality Training that was accompanied with motivational instructions in LTC elderly on lower extremity physical function and leg muscle properties. We hypothesised that an intervention program that targets motor control will effect on physical functioning and muscle strength of LTC elderly.

## Methods

### Design

**Fig. 1:**
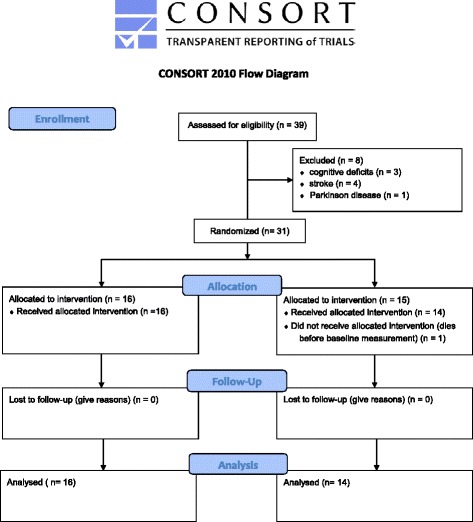
CONSORT flow chart of the study

This study follows the publication guideline of CONSORT [49], in the form of a randomised controlled trial with blinded LTC dwelling elderly individuals, randomly divided over intervention (IG) and sham control (SG) groups. The assessor and supervisors were not blinded. Measurements were carried out at baseline (BASE), after four weeks (4 W) and after eight weeks (8 W) of training. Data were collected and analysed for participants that completed 90 % of the scheduled training sessions. Figure 1 presents the study flow.

### Participants

Inclusion criteria was: age over 65 years, able to stand with or without walking aids, living in the Canton of Berne, being classified as Resident Assessment Instrument (RAI)^1^ performance level > 0, having a score ≥ 18 in the Mini-Mental Status Examination (MMSE) Test and ≤6 points on the Short Physical Performance Battery. Scoring ≤6 points on the Short Physical Performance Battery relates to poor physical performance and is a risk indicator for sarcopenia [50]. Exclusion criteria were: visual disturbances^b^, lower or upper leg prosthesis, acute joint disease, acute thrombosis, acute fractures, acute infections, acute tissue damage, acute surgical scars or alcohol abuse. The sampling frame of LTC elderly fulfilling the inclusion/exclusion criteria and receiving information about the study comprised 40 individuals (Table 1). Thirty-one LTC elderly agreed to participate and were reached through personal interview and/or public information events.

**Table 1:** Demographic characteristics at baseline (mean ± SD)

	IG	SG	*p*
Sample	16	14	
Sex (F/M)	10/6	10/4	
Age (years)	90.4 ± 6.9	87.2 ± 5.0	0.156
Age (range)	77 – 100	79 - 97	
Height (m)	1.63 ± 0.1	1.58 ± 0.1	0.256
Weight (kg)	66.9 ± 14.2	67.1 ± 20.8	0.969
BMI (kg/cm^2^)	25.1 ± 4.8	26.5 ± 6.8	0.542

*IG* intervention group, *SG* sham group, *F* female, *M* male, *BMI* Body mass index, *SPPB* Short Physical Performance Battery

### Randomisation

An independent research assistant performed randomisation, using a random Microsoft Excel 2010 table. The participants were randomly assigned to either IG or SG by means of sealed opaque envelopes distributed after the completion of all baseline assessments. Prior to the start of the intervention, written informed consent was obtained from all participants following Ethical Committee approval (Canton Berne; Registration number 147/12). The study protocol was registered at U.S. National Institute of Health (NCT02102919; https://clinicaltrials.gov/ct2/show/NCT02102919).

### Intervention protocol

All participants were familiarised with the SR-WBV and the Dance Video Game (DVG) device one week prior to the intervention period with the aim to reduce anxiety. In the DVG session a tutorial sequence was provided to ensure understanding of the task.

### Stochastic resonance whole-body vibration

Participants were exposed to SR-WBV using a Zeptor med® device (Frei Swiss AG, Zurich, Switzerland) while standing freely on both legs wearing no shoes with slight flexion of the hips, knees and ankle joints. A purpose made seat was used to allow participants to sit down during the one-minute breaks. Each intervention consisted of five one-minute vibration periods with a one-minute break between sets. The intervention took place on three days a week, over a period of eight weeks. IG vibrated with a basic frequency of 3 Hz and noise level 4 since a previous study showed this to be a good starting level for our target population [51]. To ensure progression of the exercise, the basic frequency gradually increased to 6 Hz, depending on capabilities and feedback of the individual concerned. The amplitude was a constant 4 mm during the 8 weeks period. In analogy to previously described protocols [52] progression of the exercise intensity was based on participant’s abilities related to maintain postural stability with decreasing base of support: from being able to stand with parallel positioned feet without holding onto the bars, the vibration frequency was increased gradually from 3 Hz up to 6 Hz. From 6 Hz, the parallel standing position changed to tandem standing and ended with this position while dynamic squat movements were performed on the vibrating plates. SG vibrated with a basic frequency of 1 Hz and noise level 1 with no increase of the basic frequency and no additional exercises. The 1 Hz frequency was expected to cause no training effect [53] as previously shown [54–56].

### Dance video game

As an additional cognitive element the DG performed the dance video game after the SR-WBV exercise in every training session. The dance video game was performed on metal dance pads (TX 6000 Metal DDR Platinum Pro, 93 x 14.7 x 109 cm, Mayflash Limited, Baoan Shenzhen, China) and with a specially designed modification of the StepMania (Version 3.9) free-ware [44, 57, 58]. The dance video game screen was projected on a white wall. A scrolling display of arrows moving upwards across the screen cued each move, and the participants were asked to execute the indicated steps (forward, backward, right, or left) when the arrows reached the fixed raster graphic at the top of the screen, and in time with different songs (32 to 137 beats per minute). For each training session, the participants performed for four songs of two to three minutes each, with a short break of 30 s after each song. Progression of performance was controlled through beats per minute and difficulty level and adapted based on performance of an individual.

As the levels increased, additional distracting visual cues, e.g., “bombs,” were presented. Participants had to ignore these cues and keep their attention focused on the arrows. Occasionally, some arrows were drawn-out on the target locations indicating that the participants should remain for a while on the dance pad button on one leg. The arrow sequences were generated using the Dancing Monkey MATLAB script [59] and determined step error. Electronic sensors in the dance pad detected position and timing information that was then used to provide participants with real-time visual feedback.

Video game dancing (DANCE) promotes fast, rhythmic, and accurate foot movements and may improve higher cognitive processing as measured by standard neuropsychological tests [60, 61].

### Motivation strategy

A professional exercise instructor (SR) coached the participants in both groups to enhance exercise participation and ensure that the targeted exercise frequencies and levels, sessions duration [8], and prevention of attrition [62] would be reached. The exercise instructor focused on the motivation to perform functional activities in the LTC setting [63] with the help of Motivation-Volition (MoVo) [64]. MoVo programs, where the acronym stands for “motivation” and “volition”, have previously shown to be succesfull in reaching higher compliance and fewer dropout rates for exercise in in-patient rehabilitation settings [65]. In brief, MoVo intervention programs encompass the following *motivational strategies*: (a) clarification of personal health objectives; (b) contemplation of different actions to achieve the health objectives; (c) formation of specific goal intentions; (d) checking self-concordance of this goal intention; and (f) reflection of outcome experiences. Furthermore, MoVo puts a strong emphasis on subsequent *volitional strategies*: (a) generating implementation intentions; (b) anticipating personal barriers; (c) developing counter strategies; and (d) self-monitoring the new behavior. The specific features of MoVo are described in more detail elsewhere [64].

### Primary outcome

The Short Physical Performance Battery (SPPB) [66], a standardized measure of physical performance that assesses standing balance, usual gait velocity over a 4-m course, and the time to sit down and rise from a chair five times as quickly as possible, has been recommended for use as a functional outcome measure in clinical trials in frail older persons [67]. The SPPB shows high reliability with an ICC of 0.88–0.92 [68]. SPPB scores range from 0 (*lowest function*) to 12 (*highest function*) points and can be classified as: “*weak performance*” for scores from 0 to 6 points, “*mean performance*” for 7 to 10 points, “*good performance*” for 11 to12 points [69]. The SPPB scale predicts institutionalization, hospital admission, mortality, and disability [66, 70, 71].

### Secondary outcomes

Isometric maximal voluntary contraction (IMVC) and isometric rate of force development (IRFD) of knee extension and knee flexion were measured. The onset of force was determined at 10 Newton (N) of each individual’s force-time curve. IMVC in N was determined as the maximum point on the force-time curve. Submaximal force (Fsub) values were calculated at 30, 50, 100 and 200 ms relative to the onset of force and presented the maximum voluntary contraction values (N) at this time point. IRFD was defined as the maximal slope of the force-time curve between onset of force and 200 ms (N/ms). Submaximal IRFD values (IRFDsub) were calculated as the mean slope of the force-time curve (ΔF/Δt) over time intervals of 0–30, 0–50, 0–100 and 100-200 ms relative to the onset of force [72]. The onset of force was 0 ms.

**Fig. 2:**
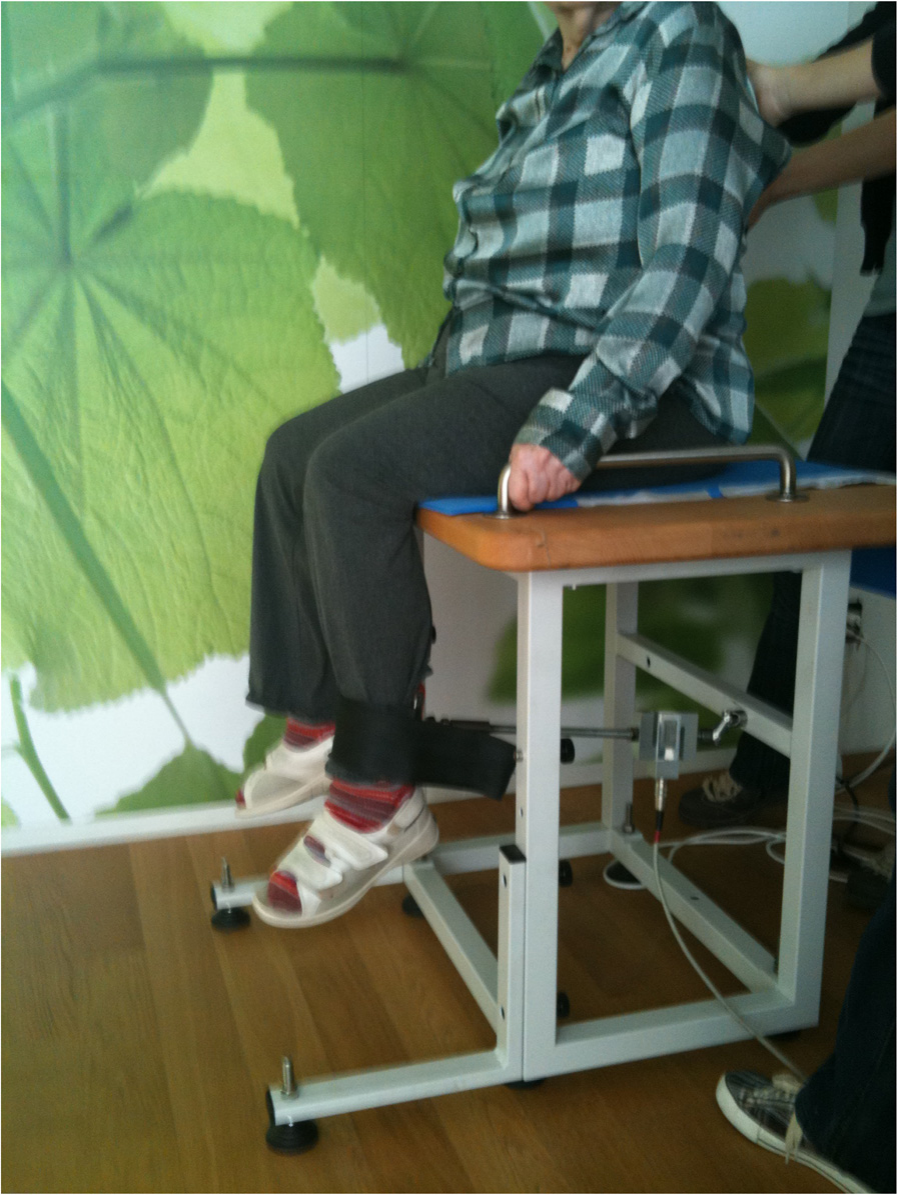
Set-up for the muscle strength assessment

A strain gauge (Sensor KM 1500S, Megatron, Munich, Germany) was used to measure the force values. The participants sat on a chair with 90° knee flexion and the dynamometers were fixed above the right (r) and left (l) ankle joint (Fig. 2). On the command “3-2-1-go!” the participants had to flex (flex) or extend (ex) each knee separately for five seconds as fast and as strongly as possible against the fixation. Details of the protocol were previously published and result in reliable measures with intraclass correlation coefficients (ICC) ranging between from 0.90 to 0.98 [73]. The analogue signal of the dynamometer was transmitted to a measurement amplifier (UMV, uk-labs, Kempen, Germany), digitalised by a 12-bit A-/D-converter (Meilhaus ME-2600i, SisNova Engineering, Zug, Switzerland) with a sampling rate of 1 kHz and analyzed with the Analogue Digital Signal processing software (ADS, uk-labs, Kempen, Germany).

### Data processing

Dynamometer data was processed with a costum-made software routine (MATLAB R2013a). After low-pass filtering (10 Hz, 2^nd^ order Butterworth, forward-backward) the force-time curves were visually inspected for plausibility and to determine the time points of force onset (10 N). Force parameters were then calculated according [72] the respective time windows.

### Sample size calculation

The primary study objective was to evaluate the effect of SR-WBV and DVG compared to sham intervention on lower extremity physical performance as assessed with the SPPB. Based on an estimated meaningful change in SPPB score of 1 point [74–76], a significance level set at 5 %, a power of 80 % to detect differences with two-sided hypothesis testing, inclusion of *N* = 30 participants (*n* = 15 per group) will be needed for a two-groups pre- post-test study design.

### Statistical analyses

Statistical Package for Social Sciences (SPSS) 22.0 for Mac (SPSS, Inc; Chicago, Illinois) was used for all statistical analyses. An intention-to-treat analysis (ITT) was performed where missing data were replaced by mean values of the group to which subjects were originally allocated [77]. Kolmogorov-Smirnov test was used to assess normality of data distribution. Baseline demographic data and baseline outcome data comparing IG and SG were analyzed by unpaired students *t*-test.

A 2 (groups) x 3 (measurements) repeated measures analysis of variance (ANOVA) was carried out to test for interactions and time effects (Greenhouse-Geisser corrected). We used nonparametric Rank-Order Tests of Puri and Sen L Statistics to assess change over time where Pillai’s Trace was used to calculate L [78–80].

For effect sizes (ES) assessing meaningfulness of differences within and between groups, eta-squared (η^2^) in ANOVA and MANOVA was used. For η^2^, an effect size of 0.01 is considered a ‘small’ effect, around 0.06 a ‘moderate’ effect and 0.14 and above a ‘large’ effect [81].

## Results

From the 40 LTC elderly approached, 31 agreed to participate (Fig. 1) resulting in a 77.5 % recruitment rate for the sampling frame. One participant from SG died before baseline measurement and eight of the 40 initially deemed eligible were willing to participate, however, did not fulfill the inclusion criteria (*n* = 3 low MMSE score; *n* = 4 with recent stroke; *n* = 1 with multiple sclerosis, *n* = 1 with parkinson disease). Training adherence rate, expressed in %; [100 ÷ (34 ÷ Mean amount of trainings visited)] revealed a mean attendance rate of 100 % (34 of 34 intervention sessions).

The participants were willing to be randomized. Neither subjective nor objective side-effects related to the used intervention were reported. At baseline, no statistically significant differences (*p* < 0.05) were found between groups.

Table 2 presents the primary and secondary outcomes at baseline. All SPPB data could be used for statistical analysis while for strength values an ITT was perfomed. The time force-curve of four participants could not be used for analysis. Fsub values and IRFDsub are listed in Additional files 1, 2, 3, 4, 5, 6, 7 and 8. At baseline no group differences were identified.

**Table 2:** SPPB ANOVA with repeated measurements (ranks) intergroup-by-time effects and group-by-time interaction

	Pillai’s trace (r^2^ = SS_Bet_/SS_Tot_)	L [(N-1) r^2^]	*p*	ES (η^2^)
SPPB Total (time effects)	0.22	0.30	0.742	0.22
SPPB Total (interaction effects)	0.60	10.20	0.001*	0.60

SPPB: Short Physical Performance Battery; °: significant difference p < 0.05, *: siginificant difference after Bonferroni adjustment *p* < 0.0125; ES: effect size (η2 = .01; small effect, η2 = .06; moderate effect, η2 = .14; large effect)

### Primary outcome: short physical performance battery

A significant interaction effect in SPPB; F(1.7,48) = 35.2, *p* < 0.001) with a large ES (η2 = 0.557) were determined in favour of IG (Table 2). The between group effect (Table 5) shows significant values after 4 weeks F(1,28 = 6.85; η2 = 0.20; *p* = 0.014) and after 8 weeks F(1,28 = 13.17; η2 = 0.32; *p* = 0.001) in favour of IG.

**Table 3:** ANOVA with repeated measurements (ranks) intergroup-by-time effects and group-by-time interaction for the secondary outcomes IMVC (N)

	Pillai’s trace (r^2^ = SS_Bet_/SS_Tot_)	L [(N-1) r^2^]	*p*	ES (η^2^)
IMVC right ex (N) (time effects)	0.001	0.006	0.994	0.001
IMVC right ex (N) (interaction effects)	0.28	5.41	0.01*	0.28
IMVC left ex (N) (time effects)	0.01	0.007	0.993	0.001
IMVC left ex (N) (interaction effects)	0.34	7.16	0.003*	0.51
IMVC right flex (N) (time effects)	0.27	1.62	0.232	0.27
IMVC right flex (N) (interaction effects)	0.001	0.003	0.997	0.001
IMVC left flex (N) (time effects)	0.15	2.54	0.097	0.15
IMVC left flex (N) (interaction effects)	0.001	0.001	0.999	0.001
	0.09	1.33	0.282	0.09

IMVC: isometric maximum voluntary contraction; °: significant difference *p* < 0.05, *: siginificant difference after Bonferroni adjustment *p* < 0.0125; ES: effect size (η2 = .01; small effect, η2 = .06; moderate effect, η2 = .14; large effect)

### Secondary outomes: strength tests

Tables 3, 4 and 5 summarise the intervention effects for the muscle strength related outcomes. IMVC showed significant changes over time for knee extension right and left and knee flexion right (Table 4). Post-hoc analysis revealed significant between group effects for knee flexion left (*p* < 0.02) after eight weeks of training (Table 5).

**Table 4:** ANOVA with repeated measurements (ranks) intergroup-by-time effects and group-by-time interaction for the secondary outcomes IRFD (N/ms)

	Pillai’s trace (r^2^ = SS_Bet_/SS_Tot_)	L [(N-1) r^2^]	*p*	ES (η^2^)
IRFD right ex (N/ms) (time effects)	0.001	0.005	0.995	0.001
IRFD right ex (N/ms) (interaction effects)	0.238	4.37	0.022*	0.24
IRFD left ex (N/ms) (time effects)	0.43	10.73	0.001*	0.43
IRFD left ex (N/ms) (interaction effects)	0.02	0.03	0.97	0.002
IRFD right flex (N/ms) (time effects)	0.68	29.38	0.001*	0.68
IRFD right flex (N/ms) (interaction effects)	1.45	6.30	0.007*	0.59
IRFD left flex (N/ms) (time effects)	0.85	52.61	0.001*	0.85
IRFD left flex (N/ms) (interaction effects)	0.52	9.65	0.001*	0.52

IRFD: isometric rate of force development; °: significant difference *p* < 0.05, *: siginificant difference after Bonferroni adjustment *p* < 0.0125; ES: effect size (η2 = .01; small effect, η2 = .06; moderate effect, η2 = .14; large effect)

**Table 5:** Between group effects at BASE, 4 W and 8 W on SPPB, IMVC and IRFD

	BASE	p/η^2^	4 W	p/η^2^	8 W	p/η^2^
SPPB (Score) (IG)	2.9 ± 1.7	0.16/0.07	5.6 ± 2.9	0.01*/0.21	7.13 ± 3.2	0.004*/0.26
SPPB (Score) (SG)	3.9 ± 1.5		3.4 ± 1.2		3.7 ± 1.2	
IMVC right ex (N) (IG)	136.0 ± 58.4	0.62/0.01	138.4 ± 56.0	0.81/0.002	180.1 ± 71.2	0.10/0.09
IMVC right ex (N) (SG)	157.9 ± 75.7		134.4 ± 59.0		147.8 ± 62.8	
IMVC left ex (N) (IG)	140.4 ± 89.3	0.97/0.001	163.1 ± 78.1	0.12/0.08	194.6 ± 90.2	0.04°/0.26
IMVC left ex (N) (SG)	132.5 ± 56.0		119.5 ± 54.0		126.1 ± 65.7	
IMVC right flex (N) (IG)	61.0 ± 29.0	0.88/0.001	74.4 ± 30.7	0.29/0.04	87.0 ± 38.5	0.08/0.11
IMVC right flex (N) (SG)	64.1 ± 31.0		67.0 ± 31.7		67.0 ± 31.2	
IMVC left flex (N) (IG)	73.5 ± 42.6	0.37/0.03	79.0 ± 44.8	0.15/0.07	86.9 ± 36.8	0.03°/0.15
IMVC left flex (N) (SG)	60.3 ± 24.5		60.4 ± 25.3		64.4 ± 16.5	
IRFD right ex (N/ms) (IG)	0.46 ± 0.3	0.70/0.005	0.56 ± 0.3	0.02*/0.06	0.72 ± 0.4	0.004*/0.25
IRFD right ex (N/ms) (SG)	0.41 ± 0.3		0.40 ± 0.2		0.39 ± 0.1	
IRFD left ex (N/ms) (IG)	0.48 ± 0.4	0.97/0.001	0.69 ± 0.5	0.02*/0.19	0.82 ± 0.5	0.001*/0.41
IRFD left ex (N/ms) (SG)	0.38 ± 0.3		0.34 ± 0.1		0.29 ± 0.1	
IRFD right flex (N/ms) (IG)	0.13 ± 0.1	0.14/0.08	0.25 ± 0.1	0.01*/0.23	0.39 ± 1.5	<0.001*/0.64
IRFD right flex (N/ms) (SG)	0.19 ± 0.1		0.15 ± 0.1		0.15 ± 0.1	
IRFD left flex (N/ms) (IG)	0.17 ± 0.1	0.97/0.001	0.26 ± 0.2	0.02*/0.19	0.39 ± 0.2	<0.001*/0.42
IRFD left flex (N/ms) (SG)	0.15 ± 0.1		0.14 ± 0.1		0.15 ± 0.1	

IG: intervention group, SG: sham group, SPPB: Short Physical Performance Battery, IMVC: isometric maximal voluntary contraction, ex: extension, flex: flexion, N: Newton, IRFD: isometric rate of force development, N/ms: NewtoN/mseconds, °: significant difference between groups *p* < 0.05, *: siginificant difference between groups after Bonferroni correction *p* < 0.025, η^2^: effect size: η^2^ = .01; small effect, η^2^ = .06; moderate effect, η^2^ = .14; large effect

Following eight weeks of SR-WBV and DVG training, Fsub measures at 30 ms, 100 ms and 200 ms in both right and left leg flexion showed a significant between group effect (*p* < 0.01) with large ES (>0.14) compared to Sham intervention (Additional files 1, 2, 3, 4, 5, 6, 7 and 8).

Table 3 shows the Greenhouse-Geisser univariate test results; a significant intragroup-by-time effect and group-by-time interaction effect following eight weeks of SR-WBV and DVG intervention on IRFD. Significant between groups effects were both seen after 4 weeks (*p* < 0.05) and 8 weeks (*p* < 0.05) in IRFD right and left leg extension and right and left leg flexion manoeuvres (Table 5).

Significant effects (*p* < 0.001) and large ES > 0.14 in the right and left leg knee extension and knee flexion movements were shown for IRFDsub at 0-30 ms, 0-50 ms, 0-100 ms and 100-200 ms (Additional files 9, 10, 11, 12, 13, 14, 15 and 16).

## Discussion

This study aimed to assess the effects of SR-WBV & Video Dance Game Training that was accompanied with motivational instructions in LTC elderly on lower extremity physical function and leg muscle properties. We hypothesised that an intervention program that targets motor control will effect on physical functioning and muscle strength levels of LTC elderly. The results of the study demonstrate that a combination of SR-WBV and DVG may be used as a skilling-up exercise for LTC elderly because of significant SPPB score change values in IG (+58.8 %) compared to SG (− 4.0 %) and concomitant significant strength improvements seen in Fsub, IRFD, and IRFDsub in IG compared to SG.

Coaching the LTC participants in both groups with a professional exercise instructor to enhance exercise participation, aimed to ensure that the targeted exercise frequencies and levels would be reached, and to prevent attrition. This approach showed to be rather succesfull. We demonstrated the feasibility of a motivational approach through high adherence rates for LTC dwelling older people randomised in this clinical trial. Our target of 75 % compliance for the 8-weeks training project was by far attained. Furthermore, no individuals were considered non-compliant for the training. Thus, compliance with the exercise interventions and retesting was excellent. Compared with median rates for recruitment, attrition and adherence in falls prevention interventions in institutional settings for clinical trials [82] we achieved better rates. However, we report on values after 8 training weeks. Nyman and Victor [82] and Fuchs and colleagues [64] report values that may be expected by 12 months. In future trials with LTC individuals the follow-up period for the assessment of adherence and attrition should, therefore, preferably be extended to a similar time frame to facilitate comparability with reference values.

Previous studies in elderly individuals have referred to the usefulness of WBV training on both muscle strength [83–87] and balance [88–91]. Playing certain types of Video Games had an effect on muscle strength [92], balance [93], and gait [94]. However, few studies found results with similar high effect sizes as this study. The combination of SR-WBV and DVG might, therefore, be more effective in activating the sensorimotor system compared to published studies that investigated solely training using one of these approaches [51, 83, 92–95]. However, future studies that compare both approaches against each other are needed to substantiate or refute this assumption. It is known that traditional strengthening improves muscle strength as a result of an improved neural drive and muscle hypertrophy [96–98]. Gruber and Gollhofer [72] postulated that sensorimotor training had a large influence on the neuromuscular system at the initiation of production of rate of force development and neuromuscular activation at the onset of voluntary actions. The motoneuron outputs induced by sensorimotor training comprise elevated central motor drive, motoneuron recruitment or firing frequency, alterations in synchronisation of motor unit firing, and reduced presynaptic inhibition [72, 99]. However, the increase in IRFD after sensorimotor training is not associated with an increase in maximum voluntary contraction [72]. As a sensorimotor training method, SR-WBV and DVG seems, therefore, to mainly affect the neural drive.

The results of the present study potentially have important functional consequences. Age-related degenerative processes, referred to as dynapenia, are considered a contributing factor to loss of independence in daily living [20]. High RFD is important in various activities of daily life where a sudden strength capacity is required, and to counteract sudden perturbations, e.g., in postural control to avoid falls [98, 100]. A typical contraction time involved in such movements is between 50 to 250 ms. In contrast, the time to reach maximum strength in most human muscles is over 300 ms, e.g., for knee extensors [101]. On the basis of the previously described reasoning it seems plausible that IMVC did not significantly change during sensorimotor training. However, physical performance improved significantly. RFD is more closely related to physical performance than IMVC [102, 103]. The result of this study is in accordance with Bottaro et al. [104], who were able to find an increase in RFD in parallel with an improvement in physical performance.

This study presents a mean change after four weeks of about 2.7 points and after eight weeks of 4.2 points on the SPPB scale in the IG. After four weeks the SG shows a mean change of about −0.5 and after eight weeks a mean change −0.2 points on the SPPB scale. Changes of about 1 point on the SPPB scale are substantial [105]. From a clinical standpoint, low SPPB point scores have a predictive value in activities in daily living [69], loss of mobility [106], admission to nursing facilities, disability [66, 69], hospitalization [107] and mortality [108]. In addition, physical performance measures have been used to test the efficacy of preventive strategies [109]. An improvement on the SPPB point scale through SR-WBV and DVG may reduce the risk of major mobility disability. Maintaining mobility is a central component in sustaining independence in daily living. Drey et al. [105] described that muscle strength and muscle power during follow up interventions have been shown to be equally beneficial for increasing physical function in elderly individuals. Future studies should be designed with adequate follow up measures to further investigate and record the possible impact of SR-WBV and DVG on the activities of daily living.

An additional advantage of this current study over previous WBV or DVG investigations in the elderly is the use of the classification system defined by Zeyfang and Braun [110]. Elderly individuals are not a homogeneous group. There are biologically elderly individuals who still feel young at heart, having a high physical fitness and performance level, and anticipating a few decades of life expectancy ahead of them. On the other spectrum frail elderly can be situated. Therefore, functional status of participating older individuals should be emphasized in training studies. To manage elderly individuals’ needs in consideration of diagnostic or treatment goals or maintenance of health, the framework “Go-Go, Slow-Go and No-Go” could be used. This classification was introduced by Zeyfang and Braun [110] classifying older adults as “being an independent person” (Go-Go); “being a needy person with a slight handicap” (Slow-Go); and “being a person in need of care with severe functional limitation” (No-Go). The need for care may be defined as depending permanently on assistance (No-Go) or depending on support in everyday activities such as dressing, body care, eating, using the toilet, mobility, and planning the day (Slow-Go) [111]. A systematic review indicated that older adults categorized in these groups react differently when provided with the same training stimuli [38]. Our study reflects the need to consider the functional status of the included participants. Bautmans et al. [88] included all residents in a nursing home within dependence categories “O” (do not need assistance), “A” (need assistance in two ADLs: washing and dressing) and “B” (require assistance in three ADLs) according to the scale of Katz et al. [112]. When their participants are categorised with our classification it becomes apparent that only Go-Go and Slow-Go elderly were included. This finding is reflective of the observation that there are only few studies [51, 83, 95, 113] that have focused separately on the Go-Go, Slow-Go and No-Go classification of older individuals.

There are some limitations in this study that should be mentioned. It was a single blind study. Studies where the examiner is not blinded might be at a higher risk of attrition [114] or assessment bias [115]. Future studies should, therefore, try to replicate our findings using a design where the examiner is blinded. No long-lasting effects follow-up measurements were obtained on the impact of the program on functional performance, strength or fall rates. Future studies should carry out such follow-up measurements to evaluate lasting effects. Furthermore, we used performance (SPPB) and impairment level (lower body muscle strength) based measures as proxies for PA [116, 117]. Although these measures are correlated and randomised clinical trial intervention studies in older adults show that PA improves measures of physical performance [109, 118] future studies should include quantified measures of PA instead of proxy measures.

## Conclusions

During SR-WBV and DVG intervention, three times a week within eight weeks physical performance and rate of force development improved significantly compared to sham intervention in No-Go (need-of-care) elderly residing in LTC. The results of this study indicate that SR-WBV and DVG can be used as a skilling up training regime for No-Go elderly individuals. Future investigations are warranted and should include follow-up measures, together with additional measures of functional strength, balance, gait and fall risk.

## Abbrevations

ADLs, activity of daily living; ANOVA, analyses of varianc; DVG, dance video game; ES, effect size; Fsub, Submaximal force; Hz, hertz; IG, intervention group; IMVC, isometric voluntary contraction; IRFD, isometric rate of force development; IRFDsub, submaximal IRFD values, Long-term care; LTC, MMSE, Mini-Mental Status Examination; ms, millisseconds; N, Newton; N/ms, Newton/milliseconds; RAI, Resident Assessment Instrument; RFD, rate of force development; SH, sham group; SPPB, Short Physival Performance Battery; SR-WBV, stochastic resonance whole-body vibration; WBV, whole-body vibration.

## Additional files

Additional file 1: ANOVA with repeated measurements (ranks) intergroup-by-time effects and group-by-time interaction for the secondary outcomes Fsub 30 ms (N). (DOCX 18 kb) Additional file 2: Outcome Fsub 30ms (N) data and between group comparison at BASE, 4 W and 8 W. (DOCX 19 kb) Additional file 3: ANOVA with repeated measurements (ranks) intergroup-by-time effects and group-by-time interaction for the secondary outcomes Fsub 50 ms (N). (DOCX 19 kb) Additional file 4: Outcome values af Fsub 50ms (N) data and between group comparison at BASE, 4 W and 8 W. (DOCX 19 kb) Additional file 5: ANOVA with repeated measurements (ranks) intergroup-by-time effects and group-by-time interaction for the secondary outcomes Fsub 100 ms (N). (DOCX 18 kb) Additional file 6: Outcome values af Fsub 100ms (N) data and between group comparison at BASE, 4 W and 8 W. (DOCX 20 kb) Additional file 7: ANOVA with repeated measurements (ranks) intergroup-by-time effects and group-by-time interaction for the secondary outcomes Fsub 2000 ms (N). (DOCX 18 kb) Additional file 8: Outcome values af Fsub 200ms (N) data and between group comparison at BASE, 4 W and 8 W. (DOCX 19 kb) Additional file 9: ANOVA with repeated measurements (ranks) intergroup-by-time effects and group-by-time interaction for the secondary outcomes IRFDsub 0-30 ms (N/ms). (DOCX 18 kb) Additional file 10: Outcome values af IRFDsub 0-30 ms (N/ms) data and between group comparison at BASE, 4 W and 8 W. (DOC 36 kb) Additional file 11: ANOVA with repeated measurements (ranks) intergroup-by-time effects and group-by-time interaction for the secondary outcomes IRFDsub 0-50 ms (N/ms). (DOC 30 kb) Additional file 12: Outcome values af IRFDsub 0-50 ms (N/ms) data and between group comparison at BASE, 4 W and 8 W. (DOC 36 kb) Additional file 13: ANOVA with repeated measurements (ranks) intergroup-by-time effects and group-by-time interaction for the secondary outcomes IRFDsub 0-100 ms (N/ms). (DOC 30 kb) Additional file 14: Outcome values af RFDsub 0-100 ms (N/ms) data and between group comparison at BASE, 4 W and 8 W. (DOC 36 kb) Additional file 15: ANOVA with repeated measurements (ranks) intergroup-by-time effects and group-by-time interaction for the secondary outcomes IRFDsub 100-200 ms (N/ms). (DOC 30 kb) Additional file 16: Outcome values af RFDsub 100-200 ms (N/ms) data and between group comparison at BASE, 4 W and 8 W. (DOC 35 kb)

## Footnotes

1 The ADL Hierarchy Scale of the RAI groups activities of daily living according to the stage of the disablement process in which they occur. Early loss ADLs (for example, dressing) are assigned lower scores than late loss ADLs (for example, eating). The ADL Hierarchy ranges from 0 (no impairment) to 6 (total dependence); source: Morris JN, Fries BE, Morris SA. (1999) Scaling ADLs within the MDS. Journals of Gerontology: Medical Sciences 54(11):M546-M553)
