# Supplementary material for: Sensory-motor training targeting motor dysfunction and muscle weakness in long-term care elderly combined with motivational strategies: a single blind randomized controlled study
Source: Eur Rev Aging Phys Act. 2016 May 28;13:4. doi: 10.1186/s11556-016-0164-0 (PMC4884400; doi:10.1186/s11556-016-0164-0)
Supplement: Additional file 2: — Outcome Fsub 30ms (N) data and between group comparison at BASE, 4 W and 8 W. (DOCX 19 kb) [file 11556_2016_164_MOESM2_ESM.docx]

### Additional file 2 – Outcome Fsub 30ms (N) data and between group comparison at BASE, 4 W and 8 W

|  | BASE | p / η^2^ | 4W | p / η^2^ | 8W | p / η^2^ |  |
| --- | --- | --- | --- | --- | --- | --- | --- |
| Fsub 30ms right ex (N) (IG) | 152.4 ± 55.1 | 0.78 / 0.003 | 138.5 ± 58 | 0.86 / 0.001 | 220.5 ± 58 | 0.022* / 019 |  |
| Fsub 30ms right ex (N/) (SG) | 166.5 ± 0.2 |  | 134.6 ± 64 |  | 153.5 ± 64.1 |  |  |
| Fsub 30ms left ex (N) (IG) | 140.6 ± 89 | 0.97 / 0.001 | 163.4 ± 78 | 0.14 / 0.08 | 195.0 ± 90 | 0.003* / 0.26 |  |
| Fsub 30ms left ex (N) (SG) | 129.8 ± 55 |  | 119.7 ± 52 |  | 121.6 ± 66 |  |  |
| Fsub 30ms right flex (N) (IG) | 61.3 ± 29 | 0.88 / 0.001 | 74.7 ± 31 | 0.29 / 0.04 | 180.6 ± 72 | 0.97 / 0.09 |  |
| Fsub 30ms right flex (N) (SG) | 64.3 ± 30.9 |  | 76.2 ± 32 |  | 141.7 ± 72 |  |  |
| Fsub 30ms left flex (N) (IG) | 73.7 ± 41 | 0.39 / 0.03 | 79.3 ± 43 | 0.16 / 0.07 | 87.3 ± 35 | 0.02* / 0.18 |  |
| Fsub 30ms left flex (N) (SG) | 58.8 ± 21 |  | 61.8 ± 22 |  | 61.3 ± 19 |  |  |

Legend: Fsub: Submaximal force, N: Newton; IG: intervention group, SG: sham group, p: between groups, ex: extension, flex: felxion, ms: milisecond, °: significant difference p < 0.05, *: siginificant difference after Bonferroni correction p < 0.025, η^2^: effect size: η^2^ = .01; small effect, η^2^ = .06; moderate effect, η^2^ = .14; large effect
