# Supplementary material for: Sensory-motor training targeting motor dysfunction and muscle weakness in long-term care elderly combined with motivational strategies: a single blind randomized controlled study
Source: Eur Rev Aging Phys Act. 2016 May 28;13:4. doi: 10.1186/s11556-016-0164-0 (PMC4884400; doi:10.1186/s11556-016-0164-0)
Supplement: Additional file 4: — Outcome values af Fsub 50ms (N) data and between group comparison at BASE, 4 W and 8 W. (DOCX 19 kb) [file 11556_2016_164_MOESM4_ESM.docx]

### Additional file 4 – Outcome values af Fsub 50ms (N) data and between group comparison at BASE, 4 W and 8 W

|  | BASE | p / η^2^ | 4W | p / η^2^ | 8W | p / η^2^ |  |
| --- | --- | --- | --- | --- | --- | --- | --- |
| Fsub 50ms right ex (N) (IG) | 136.2 ± 58 | 0.616 / 0.01 | 138.7 ± 56 | 0.55 / 0.817 | 180.7 ± 72 | 0.09 / 0.09 |  |
| Fsub 50ms right ex (N/) (SG) | 133.1 ± 75 |  | 134.6 ± 57 |  | 141.7 ± 65.2 |  |  |
| Fsub 50ms left ex (N) (IG) | 161.5 ± 88 | 0.962 / 0.001 | 163.1 ± 81 | 0.23 / 0.06 | 195.2 ± 93 | 0.01* / 0.22 |  |
| Fsub 50ms left ex (N) (SG) | 153.6 ± 56 |  | 119.8 ± 59 |  | 120.5 ± 74 |  |  |
| Fsub 50ms right flex (N) (IG) | 61.3± 29 | 0.39 / 0.25 | 87.5 ± 38 | 0.15 / 0.07 | 138.8 ± 56 | 0.84 / 0.001 |  |
| Fsub 50ms right flex (N) (SG) | 64.4 ± 31 |  | 65.8 ± 25 |  | 65.8 ± 25 |  |  |
| Fsub 50ms left flex (N) (IG) | 73.7 ± 41 | 0.88 / 0.001 | 79.4 ± 43 | 0.08 / 0.11 | 139.4 ± 56 | 0.182/ 0.002 |  |
| Fsub 50ms left flex (N) (SG) | 58.8 ± 21 |  | 61.9 ± 22 |  | 135 ± 57 |  |  |

Legend: Fsub: Submaximal force, N: Newton; IG: intervention group, SG: sham group, p: between groups, ex: extension, flex: felxion, ms: milisecond, °: significant difference p < 0.05, *: siginificant difference after Bonferroni correction p < 0.025, η^2^: effect size: η^2^ = .01; small effect, η^2^ = .06; moderate effect, η^2^ = .14; large effect
