# Supplementary material for: Sensory-motor training targeting motor dysfunction and muscle weakness in long-term care elderly combined with motivational strategies: a single blind randomized controlled study
Source: Eur Rev Aging Phys Act. 2016 May 28;13:4. doi: 10.1186/s11556-016-0164-0 (PMC4884400; doi:10.1186/s11556-016-0164-0)
Supplement: Additional file 5: — ANOVA with repeated measurements (ranks) intergroup-by-time effects and group-by-time interaction for the secondary outcomes Fsub 100 ms (N). (DOCX 18 kb) [file 11556_2016_164_MOESM5_ESM.docx]

**Addional file 5 – ANOVA with repeated measurements (ranks) intergroup-by-time effects and group-by-time interaction for the secondary outcomes Fsub 100ms (N)**

|  | **Pillai`s trace (r^2^ = SS_Bet_/SS_Tot_)** | **L [(N-1) r^2^]** | **p** | **ES (η^2^)** |
| --- | --- | --- | --- | --- |
| Fsub 100ms right ex (N) (time effects)  Fsub 100ms right ex (N) (interaction effects)  Fsub 100ms left ex (N) (time effects)  Fsub 100ms left ex (N) (interaction effects)  Fsub 100ms right flex (N) (time effects)  Fsub 100ms right flex (N) (interaction effects)  Fsub 100ms left flex (N) (time effects)  Fsub 100ms left flex (N) (interaction effects) | 0.001  0.27  0.001  0.36  0.81  0.20  0.820  0.225 | 0.006  5.29  0.008  7.36  38.77  2.29  40.98  2.61 | 0.995  0.01*  0.99  0.003*  0.001*  0.10  0.001*  0.07 | 0.001  0.27  0.001  0.34  0.81  0.20  0.82  0.23 |

Legend: Fsub: Submaximal force; °: significant difference p < 0.05, *: siginificant difference after Bonferroni adjustment p < 0.0125; ES: effect size (η2 = .01; small effect, η2 = .06; moderate effect, η2 = .14; large effect)
