# Supplementary material for: Sensory-motor training targeting motor dysfunction and muscle weakness in long-term care elderly combined with motivational strategies: a single blind randomized controlled study
Source: Eur Rev Aging Phys Act. 2016 May 28;13:4. doi: 10.1186/s11556-016-0164-0 (PMC4884400; doi:10.1186/s11556-016-0164-0)
Supplement: Additional file 6: — Outcome values af Fsub 100ms (N) data and between group comparison at BASE, 4 W and 8 W. (DOCX 20 kb) [file 11556_2016_164_MOESM6_ESM.docx]

### Additional file 6 – Outcome values af Fsub 100ms (N) data and between group comparison at BASE, 4 W and 8 W

|  | BASE | p / η^2^ | 4W | p / η^2^ | 8W | p / η^2^ |
| --- | --- | --- | --- | --- | --- | --- |
| Fsub 100ms right ex (N) (IG) | 136.3 ± 58 | 0.62 / 0.009 | 138.8 ± 56 | 0.82 / 0.002 | 180.7 ± 72 | 0.08 / 0.09 |
| Fsub 100ms right ex (N/) (SG) | 153.2 ± 0.2 |  | 134.7 ± 56 |  | 141.8 ± 65 |  |
| Fsub 100ms left ex (N) (IG) | 140.8 ± 89 | 0.97 / 0.001 | 163.6 ± 78 | 0.15 / 0.08 | 195.2 ± 90 | 0.003* / 0.26 |
| Fsub 100ms left ex (N) (SG) | 130.0 ± 55 |  | 119.9 ± 52 |  | 121.8 ± 66 |  |
| Fsub 100ms right flex (N) (IG) | 61.4 ± 29 | 0.88 / 0.001 | 87.6 ± 38 | 0.08 / 0.11 | 138.9 ± 56 | 0.82 / 0.02 |
| Fsub 100ms right flex (N) (SG) | 64.5 ± 31 |  | 65.9 ± 25 |  | 65.9 ± 25 |  |
| Fsub 100ms left flex (N) (IG) | 73.8 ± 41.1 | 0.39 / 0.03 | 79.4 ± 43 | 0.16 / 0.07 | 87.4 ± 35 | 0.01* / 0.19 |
| Fsub 100ms left flex (N) (SG) | 58.9 ± 21 |  | 62.0 ± 21 |  | 61.4 ± 19 |  |

Legend: Fsub: Submaximal force, N: Newton; IG: intervention group, SG: sham group, p: between groups, ex: extension, flex: flexion, ms: milisecond, °: significant difference p < 0.05, *: siginificant difference after Bonferroni correction p < 0.025, η^2^: effect size: η^2^ = .01; small effect, η^2^ = .06; moderate effect, η^2^ = .14; large effect
