# Supplementary material for: Sensory-motor training targeting motor dysfunction and muscle weakness in long-term care elderly combined with motivational strategies: a single blind randomized controlled study
Source: Eur Rev Aging Phys Act. 2016 May 28;13:4. doi: 10.1186/s11556-016-0164-0 (PMC4884400; doi:10.1186/s11556-016-0164-0)
Supplement: Additional file 8: — Outcome values af Fsub 200ms (N) data and between group comparison at BASE, 4 W and 8 W. (DOCX 19 kb) [file 11556_2016_164_MOESM8_ESM.docx]

### Additional file 8 – Outcome values af Fsub 200ms (N) data and between group comparison at BASE, 4 W and 8 W

|  | BASE | p / η^2^ | 4W | p / η^2^ | 8W | p / η^2^ |
| --- | --- | --- | --- | --- | --- | --- |
| Fsub 200ms right ex (N) (IG) | 136.5 ± 17 | 0.61 / 0.009 | 139.0 ± 14 | 0.82 / 0.02 | 180.8 ± 17 | 0.10 / 0.09 |
| Fsub 200ms right ex (N/) (SG) | 153.4 ± 17 |  | 134.9 ± 15 |  | 142.0 ± 17 |  |
| Fsub 200ms left ex (N) (IG) | 61.5 ± 29 | 0.88 / 0.001 | 163.7 ± 78 | 0.14 / 0.08 | 195.3 ± 90 | 0.003* / 0.26 |
| Fsub 200ms left ex (N) (SG) | 64.6± 31 |  | 120.0 ± 52 |  | 121.9 ± 66 |  |
| Fsub 200ms right flex (N) (IG) | 61.5 ± 29 | 0.88 / 0.001 | 75.0 ± 31 | 0.29 / 0.04 | 87.7 ±38 | 0.08 / 0.12 |
| Fsub 200ms right flex (N) (SG) | 64.6 ± 0.1 |  | 67.5 ± 32 |  | 66.0 ± 25 |  |
| Fsub 200ms left flex (N) (IG) | 73.9 ± 41 | 0.39 / 0.03 | 79.4 ± 43 | 0.15 / 0.07 | 87.5 ± 36 | < 0.019* / 0.18 |
| Fsub 200ms left flex (N) (SG) | 59.7 ±21.7 |  | 62.1 ± 22 |  | 61.5 ± 19 |  |

Fsub: Legend: Submaximal force, N: Newton; IG: intervention group, SG: sham group, p: between groups, ex: extension, flex: flexion, ms: milisecond, °: significant difference p < 0.05, *: siginificant difference after Bonferroni correction p < 0.025, η^2^: effect size: η^2^ = .01; small effect, η^2^ = .06; moderate effect, η^2^ = .14; large effect
