# Supplementary material for: Sensory-motor training targeting motor dysfunction and muscle weakness in long-term care elderly combined with motivational strategies: a single blind randomized controlled study
Source: Eur Rev Aging Phys Act. 2016 May 28;13:4. doi: 10.1186/s11556-016-0164-0 (PMC4884400; doi:10.1186/s11556-016-0164-0)
Supplement: Additional file 9: — ANOVA with repeated measurements (ranks) intergroup-by-time effects and group-by-time interaction for the secondary outcomes IRFDsub 0-30 ms (N/ms). (DOCX 18 kb) [file 11556_2016_164_MOESM9_ESM.docx]

**Addional file 9 – ANOVA with repeated measurements (ranks) intergroup-by-time effects and group-by-time interaction for the secondary outcomes IRFDsub 0-30ms (N/ms)**

|  | **Pillai`s trace**  **(r^2^ = SS_Bet_/SS_Tot_)** | **L [(N-1) r^2^]** | **p** | **ES (η^2^)** |
| --- | --- | --- | --- | --- |
| IRFDsub 0-30ms right ex (N/ms)(time effects)  IRFDsub 0-30ms right ex (N/ms) (interaction effects)  IRFDsub 0-30ms left ex (N/ms) (time effects)  IRFDsub 0-30ms left ex (N/ms) (interaction effects)  IRFDsub 0-30ms right flex (N/ms) (time effects)  IRFDsub 0-30ms right flex (N/ms) (interaction effects)  IRFDsub 0-30ms left flex (N/ms) (time effects)  IRFDsub 0-30ms left flex (N/ms) (interaction effects) | 0.002  0.15  0.01  0.53  0.006  0.311  0.01  0.45 | 0.03  2.07  0.17  13.37  0.07  5.41  0.12  9.78 | 0.98  0.15  0.85  0.001*  0.94  0.01*  0.89  0.001* | 0.002  0.15  0.01  0.53  0.006  0.31  0.01  0.44 |

Legend: IRFDsub: Submaximal Isometric Rate of Force Development values; °: significant difference p < 0.05, *: siginificant difference after Bonferroni adjustment p < 0.0125; ES: effect size (η2 = .01; small effect, η2 = .06; moderate effect, η2 = .14; large effect)
