# Supplementary material for: Sensory-motor training targeting motor dysfunction and muscle weakness in long-term care elderly combined with motivational strategies: a single blind randomized controlled study
Source: Eur Rev Aging Phys Act. 2016 May 28;13:4. doi: 10.1186/s11556-016-0164-0 (PMC4884400; doi:10.1186/s11556-016-0164-0)
Supplement: Additional file 11: — ANOVA with repeated measurements (ranks) intergroup-by-time effects and group-by-time interaction for the secondary outcomes IRFDsub 0-50 ms (N/ms). (DOC 30 kb) [file 11556_2016_164_MOESM11_ESM.doc]

**Additional file 11 – ANOVA with repeated measurements (ranks) intergroup-by-time effects and group-by-time interaction for the secondary outcomes IRFDsub 0-50ms (N/ms)**

|  | **Pillai`s trace**  **(r^2^ = SS_Bet_/SS_Tot_)** | **L [(N-1) r^2^]** | **p** | **ES (η^2^)** |
| --- | --- | --- | --- | --- |
| IRFDsub 0-50ms right ex (N/ms) (time effects)  IRFDsub 0-50ms right ex (N/ms) (interaction effects)  IRFDsub 0-50ms left ex (N/ms) (time effects)  IRFDsub 0-50ms left ex (N/ms) (interaction effects)  IRFDsub 0-50ms right flex (N/ms) (time effects)  IRFDsub 0-50ms right flex (N/ms) (interaction effects)  IRFDsub 0-50ms left flex (N/ms) (time effects)  IRFDsub 0-50ms left flex (N/ms) (interaction effects) | 0.001  0.26  0.001  0.45  0.001  0.46  0.001  0.19 | 0.005  4.92  0.14  13.84  0.01  12.02  0.003  3.31 | 1.00  0.02*  0.99  >0.001*  0.99  >0.001*  1.00  0.05° | 0.001  0.26  0.001  0.50  0.001  0.46  0.001  0.19 |

Legend: IRFDsub: Submaximal Isometric Rate of Force Development values; °: significant difference p < 0.05, *: siginificant difference after Bonferroni adjustment p < 0.0125; ES: effect size (η2 = .01; small effect, η2 = .06; moderate effect, η2 = .14; large effect
