# Supplementary material for: Sensory-motor training targeting motor dysfunction and muscle weakness in long-term care elderly combined with motivational strategies: a single blind randomized controlled study
Source: Eur Rev Aging Phys Act. 2016 May 28;13:4. doi: 10.1186/s11556-016-0164-0 (PMC4884400; doi:10.1186/s11556-016-0164-0)
Supplement: Additional file 12: — Outcome values af IRFDsub 0-50 ms (N/ms) data and between group comparison at BASE, 4 W and 8 W. (DOC 36 kb) [file 11556_2016_164_MOESM12_ESM.doc]

Additional file 12 – Outcome values af IRFDsub 0-50ms (N/ms) data and between group comparison at BASE, 4 W and 8 W

|  | BASE | p / η^2^ | 4W | p / η^2^ | 8W | p / η^2^ |
| --- | --- | --- | --- | --- | --- | --- |
| RFDsub 0-50ms right ex (N/ms) (IG) | 0.34 ± 0.2 | 0.77 / 0.003 | 0.63 ± 0.3 | <0.001 / 0.003 | 0.56 ± 0.2 | 0.003* / 0.27 |
| IRFDsub 0-50ms right ex (N/ms) (SG) | 0.29 ± 0.2 |  | 0.36 ± 0.3 |  | 0.25 ± 0.1 |  |
| IRFDsub 0-50ms left ex (N/ms) (IG) | 0.31 ± 0.3 | 0.24 / 0.05 | 0.53 ± 0.3 | 0.003* / 0.27 | 0.75 ± 0.4 | 0.001* / 0.33 |
| IRFDsub 0-50ms left ex (N/ms) (SG) | 0.32 ± 0.1 |  | 0.25 ± 0.2 |  | 0.30 ± 0.2 |  |
| IRFDsub 0-50ms right flex (N/ms) (IG) | 0.10± 0.05 | 0.44 / 0.02 | 0.6 ± 0.1 | <0.001* / 0.43 | 0.39 ± 2 | < 0.001* / 0.59 |
| IRFDsub 0-50ms right flex (N/ms) (SG) | 0.12 ± 0.07 |  | 0.10 ± 0.04 |  | 0.11 ± 0.05 |  |
| IRFDsub 0-50ms left flex (N/ms) (IG) | 0.13 ± 0.1 | 0.67 / 0.006 | 0.29 ± 0.2 | 0.003* / 0.27 | 0.42 ± 0.2 | < 0.001* / 0.41 |
| IRFDsub 0-50ms left flex (N/ms) (SG) | 0.09 ± 0.05 |  | 0.10 ± 0.06 |  | 0.10 ± 0.06 |  |

Legend: IRFDsub: Submaximal Isometric Rate of Force Development values, N: Newton; IG: intervention group, SG: sham group, p: between groups, ex: extension, flex: flexion, ms: milisecond, °: significant difference p < 0.05, *: siginificant difference after Bonferroni correction p < 0.025, η^2^: effect size: η^2^ = .01; small effect, η^2^ = .06; moderate effect, η^2^ = .14; large effect
