# Supplementary material for: Sensory-motor training targeting motor dysfunction and muscle weakness in long-term care elderly combined with motivational strategies: a single blind randomized controlled study
Source: Eur Rev Aging Phys Act. 2016 May 28;13:4. doi: 10.1186/s11556-016-0164-0 (PMC4884400; doi:10.1186/s11556-016-0164-0)
Supplement: Additional file 15: — ANOVA with repeated measurements (ranks) intergroup-by-time effects and group-by-time interaction for the secondary outcomes IRFDsub 100-200 ms (N/ms). (DOC 30 kb) [file 11556_2016_164_MOESM15_ESM.doc]

**Additional file 15 – ANOVA with repeated measurements (ranks) intergroup-by-time effects and group-by-time interaction for the secondary outcomes IRFDsub 100-200ms (N/ms)**

|  | **Pillai`s trace**  **(r^2^ = SS_Bet_/SS_Tot_)** | **L [(N-1) r^2^]** | **p** | **ES (η^2^)** |
| --- | --- | --- | --- | --- |
| IRFDsub 100-200ms right ex (N/ms) (time effects)  IRFDsub 100-200ms right ex (N/ms) (interaction effects)  IRFDsub 100-200ms left ex (N/ms) (time effects)  IRFDsub 100-200ms left ex (N/ms) (interaction effects)  IRFDsub 100-200ms right flex (N/ms) (time effects)  IRFDsub 100-200ms right flex (N/ms) (interaction effects)  IRFDsub 100-200ms left flex (N/ms) (time effects)  IRFDsub 100-200ms left flex (N/ms) (interaction effects) | 0.01  0.29  0.02  0.37  0.003  0.43  0.02  0.32 | 0.15  5.26  0.22  7.60  0.04  9.70  0.26  6.10 | 0.87  0.01*  0.80  0.003*  0.96  0.001*  0.77  0.007* | 0.01  0.29  0.02  0.37  0.003  0.43  0.02  0.38 |

Legend: IRFDsub: Submaximal Isometric Rate of Force Development values; °: significant difference p < 0.05, *: siginificant difference after Bonferroni adjustment p < 0.0125; ES: effect size (η2 = .01; small effect, η2 = .06; moderate effect, η2 = .14; large effec
