# Supplementary material for: Sensory-motor training targeting motor dysfunction and muscle weakness in long-term care elderly combined with motivational strategies: a single blind randomized controlled study
Source: Eur Rev Aging Phys Act. 2016 May 28;13:4. doi: 10.1186/s11556-016-0164-0 (PMC4884400; doi:10.1186/s11556-016-0164-0)
Supplement: Additional file 16: — Outcome values af RFDsub 100-200 ms (N/ms) data and between group comparison at BASE, 4 W and 8 W. (DOC 35 kb) [file 11556_2016_164_MOESM16_ESM.doc]

### Additional file 16 – Outcome values af RFDsub 100-200ms (N/ms) data and between group comparison at BASE, 4 W and 8 W

|  | BASE | p / η^2^ | 4W | p / η^2^ | 8W | p / η^2^ |
| --- | --- | --- | --- | --- | --- | --- |
| IRFDsub 100-200ms right ex (N/ms) (IG) | 0.28 ± 0.2 | 0.92 / 0.01 | 0.39 ± 0.2 | 0.005* / 0.26 | 0.56 ± 0.3 | < 0.003* / 0.30 |
| IRFDsub 100-200ms right ex (N/ms) (SG) | 0.27 ± 0.1 |  | 0.21 ± 0.1 |  | 0.28 ± 0.1 |  |
| IRFDsub 100-200ms left ex (N/ms) (IG) | 0.27 ± 0.3 | 0.94 / 0.01 | 0.45 ± 0.3 | 0.003* / 0.29 | 0.65 ± 0.2 | < 0.001* / 0.61 |
| IRFDsub 100-200ms left ex (N/ms) (SG) | 0.21 ± 0.1 |  | 0.21 ± 0.1 |  | 0.27 ± 0.1 |  |
| IRFDsub 100-200ms right flex (N/ms) (IG) | 0.08 ± 0.04 | 0.32 / 0.04 | 0.32 ± 0.2 | < 0.001* / 0.50 | 0.46 ± 0.2 | < 0.001* / 0.54 |
| IRFDsub 100-200ms right flex (N/ms) (SG) | 0.10 ± 0.08 |  | 0.11 ± 0.1 |  | 0.11 ± 0.1 |  |
| IRFDsub 100-200ms left flex (N/ms) (IG)  IRFDsub 100-200ms left flex (N/ms) (IG) | 0.17 ± 0.2  0.10 ± 0.08 | 0.77 / 0.03 | 0.39 ± 0.2  0.08 ± 0.6 | < 0.001* / 0.48 | 0.60 ± 0.2  0.09 ± 0.06 | < 0.001* / 0.70 |

LegeLegend: IRFDsub: Submaximal Isometric Rate of Force Development values, N: Newton; IG: intervention group, SG: sham group, p: between groups, ex: extension, flex: flexion, ms: milisecond, °: significant difference p < 0.05, *: siginificant difference after Bonferroni correction p < 0.025, η^2^: effect size: η^2^ = .01; small effect, η^2^ = .06; moderate effect, η^2^ = .14; large effect
